# Knowledge, Attitudes, and Practices of General Practitioners and Nonrheumatologists on Rheumatoid Arthritis in Burkina Faso in 2024: A Cross‐Sectional Study

**DOI:** 10.1002/hsr2.71099

**Published:** 2025-07-18

**Authors:** Ismael Ayouba Tinni, Wendyam Nadège Yameogo, Yannick Laurent Tchenadoyo Bayala, Bakoubassé Aissata Son, Issa Ouedraogo, Fulgence Kabore, Wendlassida Joelle Stéphanie Zabsonre/Tiendrebeogo, Dieu‐Doné Ouedraogo

**Affiliations:** ^1^ Rheumatology Department Bogodogo University Hospital Ouagadougou Burkina Faso; ^2^ Joseph KI‐ZERBO University Ouagadougou Burkina Faso

**Keywords:** attitudes, Burkina Faso, knowledge, practices, rheumatoid arthritis

## Abstract

**Background and Aims:**

Rheumatoid arthritis is still poorly understood by medical personnel in sub‐Saharan Africa, resulting in significant diagnostic delays. The aim of this study was to assess the knowledge, attitudes and practices of general practitioners and nonrheumatologist specialists regarding rheumatoid arthritis.

**Methods:**

This was a descriptive and analytical cross‐sectional study from January 22 to February 22, 2024. Data were collected using an online self‐administered questionnaire from general practitioners and nonrheumatology specialists practicing in Burkina Faso. The association between variables was tested using the chi‐squared test. The significance level was set at 5%.

**Results:**

One hundred and forty‐five physicians completed the questionnaire. The mean age of the participants was 32.01 ± 4.066 [25; 47] years. The proportion of men was 62.10% (90). The proportion of general practitioners was 61.40% (89). The mean knowledge score was 12.51 ± 2.741 [4; 18] out of 20. Of the 145 physicians who completed the questionnaire, 22.76% (33) were confronted with rheumatoid arthritis in their practice. The mean attitude score was 9.64 ± 3.389 (3 − 16) out of 20. The mean practice score was 14.76 ± 2.292 (9 − 19) out of 20. Physicians with average/good knowledge had no idea about biotherapy in 72.90% (62) of the cases, compared to 27.10% (23) who had an idea about biotherapy (*p* = 0.001). Physicians with average/good knowledge had unfavorable attitudes in 58.30% (14) of cases compared to 41.70% (10) who had favorable attitudes (*p* = 0.42). Physicians with average/good knowledge had inadequate practices in 29.20% (7) of cases versus 70.80% (17) with adequate practices (*p* = 0.019).

**Conclusion:**

The majority of participants had an acceptable level of knowledge, attitude and practice regarding rheumatoid arthritis.

## 1 Introduction

Rheumatoid arthritis (RA) is a systemic autoimmune disease that primarily affects the joints, resulting in joint pain and destruction, as well as extracellular manifestations [1]. The prevalence of rheumatoid arthritis varies from country to country, with a generally higher prevalence in developed countries, which may be explained not only by exposure to environmental risk factors, but also by genetic factors and demographic differences [1, 2]. The disease appears to be less common in Africa and Asia than in the United States and Europe [3]. The global prevalence of RA has been estimated to be between 0.24% and 1%, although rates vary between countries and regions [4]. In 2017, the Global Burden of Disease study showed an increase of 8.20% in the annual incidence rate and 7.40% in the global age‐standardized prevalence of RA compared to 1990. In Burkina Faso, the hospital incidence of RA is 1.84% of all patients seen in rheumatology consultations [5]. However, in sub‐Saharan Africa in general, and in Burkina Faso in particular, RA is still poorly understood by health professionals, resulting in significant diagnostic delays. This delay in diagnosis is responsible for joint destruction, leading to functional disability and reduced quality of life for patients [6]. In addition, the physician is usually the first point of access to a quality healthcare system. As with other diseases, their role is crucial in the early diagnosis, treatment, and follow‐up of RA patients [7]. There are no studies in Burkina Faso assessing the level of knowledge of rheumatoid arthritis among physicians. The aim of this study was to assess the knowledge, attitudes, and practices of general practitioners and nonrheumatology specialists regarding rheumatoid arthritis.

## 2 Methods

### 2.1 Study Design, Period, Population, and Area

Under the supervision of the Burkinabe Society of Rheumatology (BSR), we conducted a questionnaire survey among general practitioners and nonrheumatology specialists practicing in the public and private sectors in all regions of Burkina Faso.

This was a descriptive and analytical cross‐sectional study conducted from January 22 to February 22, 2024. Data were collected using a self‐administered online questionnaire covering epidemiologic, clinical, paraclinical and therapeutic aspects of rheumatoid arthritis.

### 2.2 Data Collection Tools and Procedure

The questionnaire was developed by the BSR, based on existing literature and the expertise of its members. Its content validity was assessed by a panel of experts. A pilot test was then conducted with physicians who were not specialists in rheumatology, to evaluate the clarity and relevance of the items before the start of data collection. Adjustments were made based on the feedback received. The survey was self‐administered and anonymous. The access link to the questionnaire was distributed through standard professional channels (emails, medical discussion groups). It is available on the KoboCollect platform at the following address: https://ee.kobotoolbox.org/preview/oifK51vm. Participation was voluntary, and informed consent was obtained online before beginning the questionnaire.

The questionnaire consisted of multiple‐choice items. It consists of four parts:

The first part is designed to collect information on socio‐professional aspects of general practitioners and nonrheumatology specialists.

The second part was divided into 12 questions assessing the knowledge of general practitioners and nonrheumatologists about epidemiologic, clinical, paraclinical and therapeutic aspects of rheumatoid arthritis. Each correct answer was worth 1 point, and a final score of 20 points was given to each participant. The ESSI et al scale was used to determine the level of knowledge [8]. Knowledge was then classified as poor if less than 50% correct, insufficient if less than 65% correct, average if less than 85% correct and good if more than 85% correct [8].

The third part was divided into 12 questions assessing the attitudes of general practitioners and nonrheumatology specialists towards RA. Each correct answer was worth 1 point, and a final score of 20 points was given to each participant. The ESSI et al. scale was used to determine the level of attitudes [8]. Attitudes were then classified as unfavorable if less than 65% correct and favorable if more than 65% correct [8].

The fourth part was divided into 10 questions assessing the practices of general practitioners and nonrheumatologists regarding the diagnostic methods for RA, the therapeutic approach to a patient with RA, and the indices for monitoring RA. Each correct answer was worth 1 point, and a final score of 20 points was given to each participant. The ESSI et al scale was used to determine the level of practice [8]. Practices were then classified as inadequate if less than 65% correct and as adequate if more than 65% correct [8].

### 2.3 Data Processing and Analysis

The questionnaire was designed and distributed using the KoboCollect platform. The collected data were analyzed using SPSS version 20 software. Quantitative variables were expressed as mean ± standard deviation and median, according to the distribution curve (symmetrical or not). Qualitative variables were expressed as percentages and numbers. The association between the dependent variables (knowledge, attitudes, and practices) and the independent variables was tested using the chi‐square test (comparison of proportions), with a significance threshold of 5%.

### 2.4 Ethical Consideration

The study protocol received authorization from the institutional ethics committee of the Bogodogo university hospital center, by deliberation N° 2023‐01‐0072A. Administrative authorization for data collection was also obtained. Confidentiality and anonymity were respected during data collection and processing, in accordance with the Helsinki recommendations. Consent was obtained from all participants.

## 3 Results

One hundred and forty‐five physicians responded to the questionnaire out of 5862 registered with the Burkina Faso Medical Association as of September 2023, representing a response rate of 2.47% (ordremedecinsburkina.bf).

The mean age of the participants was 32.01 ± 4.066 [25; 47] years. The proportion of men was 62.10% (90), with a sex ratio of 1.64. General practitioners accounted for 61.40% (89) of the participants. Participants were employed: 52.40% (76) in a university hospital (CHU), 69.70% (101) in a public facility, and 66.20% (96) were trained at the Joseph KI‐ZERBO University of Ouagadougou. The mean time in practice was 4.74 ± 3.291 [1; 19] years. The mean knowledge score was 12.51 ± 2.741 [4; 18] out of 20. Table 1 shows the distribution of participants according to socio‐professional aspects and knowledge during the study period.

**Table 1:** Distribution of participants according to socio‐professional aspects and knowledge during the study period.

Variables	Number	Percentage
Age range		
≤ 32 years	58	40
> 32 years	87	60
Physician		
Generalist	89	61.40
Nonrheumatologist specialist	56	38.60
Residence		
Urban	133	91.70
Rural	12	8.30
Place of work		
University Hospital Center	76	52.40
Private sector	23	15.90
Regional Hospital Center	21	14.50
Medical Center	14	9.70
Medical Center with Surgical Unit	11	7.50
Activity mode		
Public	101	69.70
Public and private	26	17.90
Private	18	12.40
University of training		
Joseph KI‐ZERBO University of Ouagadougou	96	66.20
Saint Thomas Aquin University of Ouagadougou	20	13.80
NAZI BONI University of Bobo Dioulasso	18	12.40
Others*	11	7.60
Practice time		
≤ 5 years	98	67.60
> 5 years	47	32.40
RA is the most common inflammatory rheumatic disease		
Yes	113	77.90
No	32	22.10
Notion about biotherapy		
Yes	118	81.40
No	27	18.60
Level of knowledge		
Bad	21	14.50
Insufficient	39	26.90
Average	77	51.10
Good	8	5.50

Abbreviation: RA, rheumatoid arthritis.

* University of Ouahigouya, University Abdou Moumouni of Niamey, University Cheik Anta Diop of Dakar, University Felix Houphouet Boigny of Abidjan.

Of the 145 physicians who answered the questionnaires, 22.76% (33) were confronted with rheumatoid arthritis in their practice. The mean age was 32.82 ± 3.33 [26; 40] years, and 69.70% (23) were men, resulting in a sex ratio of 2.3. The mean exercise duration was 5.15 ± 2.526 [1; 11] years. The mean attitude score was 9.64 ± 3.389 [3; 16] out of 20. The mean practice score was 14.76 ± 2.292 [9; 19] out of 20. Table 2 shows the distribution of participants who were confronted with rheumatoid arthritis in practice according to socio‐professional aspects, attitudes, and practices during the study period.

**Table 2:** Distribution of participants confronted with rheumatoid arthritis according to socio‐professional aspects, attitudes and practices during the study period.

Variables	Number	Percentage
Age range		
≤ 32 years	10	30.30
> 32 years	23	69.70
Physician		
Generalist	21	63.60
Nonrheumatologist specialist	12	36.40
Residence		
Urban	28	84.80
Rural	5	15.20
Activity mode		
Public	24	72.70
Public and private	8	24.30
Private	1	30
University of training		
Joseph KI‐ZERBO University of Ouagadougou	20	60.60
Saint Thomas Aquin University of Ouagadougou	4	12.10
NAZI BONI University of Bobo Dioulasso	3	9.10
Others*	6	18.20
Practice time		
≤ 5 years	20	60.60
> 5 years	13	39.40
Attitude levels		
Unfavorable	21	63.60
Favorable	12	36.40
How are you confronted with RA?		
In the context of exploring joint complaints	23	69.70
For follow‐ups diagnosed by another practitioner	10	30.30
In the context of RA‐related pathologies	8	24.20
In the case of monitoring. which we have diagnosed ourselves	8	24.20
Collaboration with a rheumatologist		
Yes	20	60.6
No	13	39.4
Level of practice	77	51.10
Inadequate	14	42.40
Adequate	19	57.60
Search for a family history of RA in cases of suspected RA		
Yes	28	84.8
No	5	15.2
Prescription of a pre‐therapeutic assessment before starting background treatment		
Yes	23	69.7
No	10	30.3
Proposing contraception to patients before starting background treatment		
Yes	20	60.6
No	13	39.4

Abbreviation: RA, rheumatoid arthritis.

* University of Ouahigouya, University Abdou Moumouni of Niamey, University Cheik Anta Diop of Dakar, University Felix Houphouet Boigny of Abidjan.

Physicians with average/good knowledge had no idea about biotherapy in 72.90% (62) of cases versus 27.10% (23) who had an idea about biotherapy (*p* = 0.001). Physicians with average/good knowledge had unfavorable attitudes in 58.30% (14) of cases versus 41.70% (10) who had favorable attitudes (*p* = 0.42). Physicians with average/good knowledge had inadequate practices in 29.20% (7) of cases versus 70.80% (17) with adequate practices (*p* = 0.019). Physicians with favorable attitudes collaborated with a rheumatologist in 91.70% (11) of cases versus 8.30% (1) who did not collaborate with a rheumatologist (*p* = 0.007). In 94.70% (18) of cases, physicians with adequate practices ordered a pretreatment evaluation before starting disease‐modifying therapy, compared to 5.30% (1) who did not (*p* < 0.001). In 79% (15) of cases, physicians with adequate practices offered contraception before starting background therapy, compared with 21% (4) who did not offer contraception before starting background therapy (*p* = 0.015). Table 3 shows the results of the bivariate analysis between level of knowledge, level of attitudes, level of practices, and socio‐professional aspects of the participants during the study period.

**Table 3:** bivariate analysis of participants' level of knowledge, level of attitudes, level of practices, and socio‐professional aspects during the study period.

Variables	Knowledge (145)			Attitudes (33)			Practices (33)		
	Bad/Insufficient *N* (%)	Average/Good *N* (%)	*p* value	Unfavorable *N* (%)	Favorable *N* (%)	*p* value	Inadequate *N* (%)	Adequate *N* (%)	*p* value
Gender									
Female	25 (45.50)	30 (54.50)	0.43	6 (60)	4 (40)	0.53	3 (30)	7 (70)	0.28
Male	35 (38.90)	55 (61.10)		15 (65.20)	8 (34.80)		11 (47.80)	12 (52.20)	
Age range									
≤ 32 years	21 (36.20)	37 (63.80)	0.30	7 (70)	3 (30)	0.46	5 (50)	5 (50)	0.41
> 32 years	39 (44.80)	48 (55.20)		14 (60.90)	9 (39.10)		9 (39.10)	14 (60.90)	
Residence									
Rural	4 (33.30)	8 (66.70)	0.55	17 (60.70)	11 (39.30)	0.38	12 (42.90)	16 (57.10)	0.64
Urban	56 (42.10)	77 (57.90)		4 (80)	1 (20)		2 (40)	3 (60)	
Activity mode			0.36			0.23			0.29
Public	41 (40.60)	60 (59.40)		1 (50)	1 (50)		1 (50)	1 (50)	
Private	10 (55.60)	8 (44.40)		7 (30.40)	16 (69.60)		11 (47.80)	12 (52.20)	
Public and private	9 (34.60)	17 (65.40)		4 (50)	4 (50)		2 (25)	6 (75)	
Practice time			0.57			0.43			0.49
≤ 5 years	39 (39.80)	59 (60.20)		12 (60)	8 (40)		9 (45)	11 (55)	
> 5 years	21 (44.70)	26 (55.30)		9 (69.20)	4 (30.80)		5 (38.50)	8 (61.50)	
Physician									
Generalist	38 (42.70)	51 (57.30)	0.68	14 (66.70)	7 (33.30)	0.45	10 (47.60)	11 (52.40)	0.33
Nonrheumatologist specialist	22 (39.30)	34 (60.70)		7 (58.30)	5 (41.70)		4 (60)	8 (60)	
University of training			0.43			0.94			0.92
Joseph KI‐ZERBO University of Ouagadougou	40 (41.70)	56 (58.30)		7 (35)	13 (65)		9 (45)	11 (55)	
Saint Thomas Aquin University of Ouagadougou	7 (35)	13 (75)		2 (50)	2 (50)		2 (50)	2 (50)	
NAZI BONI University of Bobo Dioulasso	10 (55.60)	8 (44.40)		1 (33.30)	2 (66.70)		1 (33.30)	2 (66.70)	
Others*	3 (27.30)	8 (72.70)		2 (33.30)	4 (66.70)		2 (33.30)	4 (66.70)	

* University of Ouahigouya, University Abdou Moumouni of Niamey, University Cheik Anta Diop of Dakar, University Felix Houphouet Boigny of Abidjan.

## 4 Discussion

The aim of this study was to assess the knowledge, attitudes, and practices of general practitioners and nonrheumatologist specialists regarding rheumatoid arthritis.

The mean knowledge score was 12.51/20. The majority (51.10%) of the participants had an average level of knowledge about rheumatoid arthritis. The level of knowledge was not associated with the socio‐professional aspects of the participants. In our series, the majority of participants had an average level of knowledge. This result is similar to that reported in Morocco [9]. In Egypt, physicians had a high level of knowledge about rheumatoid arthritis [10]. The average attitude score was 9.64. The majority (63.60%) of participants had unfavorable attitudes towards rheumatoid arthritis. The level of attitude was not associated with the participants' level of knowledge or socio‐professional aspects. These results may be explained by the physicians' level of knowledge about rheumatoid arthritis, although it was not statistically associated with attitudes. In addition, physicians who were confronted with RA in practice were young, with a mean age of 32.82 years and a mean time in practice of 5.15 years. The mean practice score was 14.76. The majority (57.60%) of participants had adequate rheumatology practice. There was an association between the level of practice and the level of knowledge. However, there was no association between the level of practice and the socio‐professional aspects of the participants. This result may be explained by the fact that the majority (60.60%) of participants who were confronted with RA were trained at the Joseph KI‐ZERBO University in Ouagadougou, where teaching in rheumatology has been provided by doctors specializing in rheumatology since 2012.

The level of knowledge was not associated with the attitudes of the participants. However, there was an association with the level of practice. These differences may be explained by the fact that the questionnaire was administered online. In addition, a practice analysis questionnaire administered to practitioners is not an accurate reflection of actual practice. Furthermore, it has been shown that practitioners do not always have the same attitude toward a written case as to a simulated one [9].

In our series, the majority (69.70%) of the physicians worked in a public institution. In Morocco, 54.10% of physicians worked in the public sector in a survey on the management of rheumatoid arthritis by physicians [9]. In Tunisia, 32% of physicians worked in public hospitals in a survey of physicians' attitudes towards chronic inflammatory rheumatism [7]. This rate can be explained by the fact that in Africa in general and in Burkina Faso in particular, the main employer of physicians is the state. In addition, the private sector is not very developed in Burkina Faso, and the various private structures are not obliged to recruit permanent doctors.

The majority of physicians (69.70%) were confronted with RA during the evaluation of joint complaints and 30.30% during the follow‐up of a diagnosis made by another physician. In Tunisia, 26% of physicians were confronted with chronic inflammatory rheumatism in the context of the investigation of joint complaints and 21% in the context of the follow‐up of chronic inflammatory rheumatism diagnosed by another physician [7]. These differences may be explained by the fact that the Tunisian study included 57 physicians and focused on chronic inflammatory rheumatism in general.

Our study has limitations. The participation rate was only 2.47%, which may lead to selection bias. In addition, the questionnaire was administered online, which may lead to response bias. Any interpretation of our results must take these limitations into account.

## 5 Conclusion

The majority of participants had an acceptable level of knowledge, attitude, and practice regarding rheumatoid arthritis. However, these different levels could be improved through postgraduate teaching, training workshops, and the introduction of a university diploma in the diagnosis and management of RA by the Burkinabe Rheumatology Society to enable better patient management.

## Author Contributions

**Ismael Ayouba Tinni:** conceptualization, methodology, data curation, investigation, writing – original draft, formal analysis, validation. **Wendyam Nadège Yameogo:** conceptualization, data curation, methodology, investigation, writing – original draft, formal analysis, validation. **Yannick Laurent Tchenadoyo Bayala:** conceptualization, data curation, formal analysis, writing – original draft, methodology, investigation, validation. **Bakoubassé Aissata Son:** conceptualization, data curation, formal analysis, writing – original draft, methodology, investigation, validation. **Issa Ouedraogo:** data curation, conceptualization, formal analysis, writing – original draft, methodology, investigation, validation. **Fulgence Kabore:** validation, writing – review and editing, methodology, supervision, formal analysis, visualization. **Wendlassida Joelle Stéphanie Zabsonre/Tiendrebeogo:** methodology, validation, formal analysis, supervision, visualization. **Dieu‐Doné Ouedraogo:** methodology, supervision, formal analysis, validation, writing – review and editing, visualization.

## Ethics Statement

All authors have read and approved the final version of the manuscript. Ayouba Tinni Ismael had full access to all of the data in this study and takes complete responsibility for the integrity of the data and the accuracy of the data analysis.

## Conflicts of Interest

The authors declare no conflicts of interest.

## Transparency Statement

The lead author Ismael Ayouba Tinni affirms that this manuscript is an honest, accurate, and transparent account of the study being reported; that no important aspects of the study have been omitted; and that any discrepancies from the study as planned (and, if relevant, registered) have been explained.

## Data Availability

The data that support the findings of this study are available from the corresponding author upon reasonable request.
